# Supplementary material for: Luteolin Protects Pancreatic β Cells against Apoptosis through Regulation of Autophagy and ROS Clearance
Source: Pharmaceuticals (Basel). 2023 Jul 7;16(7):975. doi: 10.3390/ph16070975 (PMC10385282; doi:10.3390/ph16070975)
Supplement: Supplementary file 1 [file pharmaceuticals-16-00975-s001.zip › pharmaceuticals-2289062-supplementary.pdf]

Table S1. Effect of screened compounds on DRAK2 activity at a concentration of 20 μM

| No. | Concentration | Result Type | Result |
|---|---|---|---|
| JY-01519(Luteolin) | 20 μM | %Activity | 7.74 +/- 1.75 |
| JY-01522 | 20 μM | %Activity | 94.17 +/- 6.84 |
| JY-01523 | 20 μM | %Activity | 66.57 +/- 5.02 |
| JY-01524 | 20 μM | %Activity | 90.62 +/- 14.87 |
| JY-01526 | 20 μM | %Activity | 54.19 +/- 1.74 |
| JY-01527 | 20 μM | %Activity | 66.35 +/- 6.03 |
| JY-01529 | 20 μM | %Activity | 68.73 +/- 7.78 |
| JY-01530 | 20 μM | %Activity | 79.49 +/- 4.96 |
| JY-01531 | 20 μM | %Activity | 64.85 +/- 1.14 |
| JY-01532 | 20 μM | %Activity | 71.04 +/- 6.00 |
| JY-01536 | 20 μM | %Activity | 89.42 +/- 5.85 |
| JY-01540 | 20 μM | %Activity | 37.86 +/- 6.00 |
| JY-01541 | 20 μM | %Activity | 57.94 +/- 4.99 |
| JY-01542 | 20 μM | %Activity | 62.09 +/- 4.02 |
| JY-01543 | 20 μM | %Activity | 82.72 +/- 3.50 |
| JY-01545 | 20 μM | %Activity | 58.23 +/- 0.59 |
| JY-01546 | 20 μM | %Activity | 50.13 +/- 8.84 |
| JY-01547 | 20 μM | %Activity | 42.79 +/- 1.24 |
| JY-01548 | 20 μM | %Activity | 67.75 +/- 2.59 |
| JY-01549 | 20 μM | %Activity | 39.83 +/- 3.97 |
| JY-01550 | 20 μM | %Activity | 41.67 +/- 0.74 |
| JY-01551 | 20 μM | %Activity | 46.50 +/- 0.31 |
| JY-01552 | 20 μM | %Activity | 70.31 +/- 0.46 |
| JY-01553 | 20 μM | %Activity | 56.54 +/- 1.82 |
| JY-01554 | 20 μM | %Activity | 64.38 +/- 10.76 |
| JY-01555 | 20 μM | %Activity | 62.46 +/- 10.21 |
| JY-01556 | 20 μM | %Activity | 75.10 +/- 7.60 |
| JY-01558 | 20 μM | %Activity | 35.54 +/- 5.27 |
| JY-02089 | 20 μM | %Activity | 132.98 +/- 8.06 |
| JY-02090 | 20 μM | %Activity | 99.30 +/- 17.26 |
| JY-02091 | 20 μM | %Activity | 114.00 +/- 3.33 |
| JY-02092 | 20 μM | %Activity | 100.80 +/- 3.01 |
| JY-02093 | 20 μM | %Activity | 100.85 +/- 3.11 |
| JY-02094 | 20 μM | %Activity | 94.30 +/- 8.89 |
| JY-02095 | 20 μM | %Activity | 96.97 +/- 12.62 |
| JY-02096 | 20 μM | %Activity | 87.74 +/- 4.46 |
| JY-02097 | 20 μM | %Activity | 87.24 +/- 3.42 |
| JY-02098 | 20 μM | %Activity | 101.29 +/- 9.46 |
| JY-02099 | 20 μM | %Activity | 121.20 +/- 4.76 |
| JY-02100 | 20 μM | %Activity | 107.05 +/- 14.73 |
| JY-02101 | 20 μM | %Activity | 124.97 +/- 1.60 |
| JY-02102 | 20 μM | %Activity | 117.22 +/- 1.77 |
| JY-02103 | 20 μM | %Activity | 120.93 +/- 1.52 |
| JY-02104 | 20 μM | %Activity | 90.94 +/- 5.62 |
| JY-02105 | 20 μM | %Activity | 115.96 +/- 2.70 |
| JY-02106 | 20 μM | %Activity | 91.04 +/- 10.28 |
| JY-02107 | 20 μM | %Activity | 84.23 +/- 5.15 |
| JY-02108 | 20 μM | %Activity | 77.85 +/- 8.63 |

**Table S1** ***Related to Figure 1.*** **Effect of screened natural products on DRAK2 activity**

The kinase activity of DRAK2 after treatment with natural product at a concentration of 20 μM.

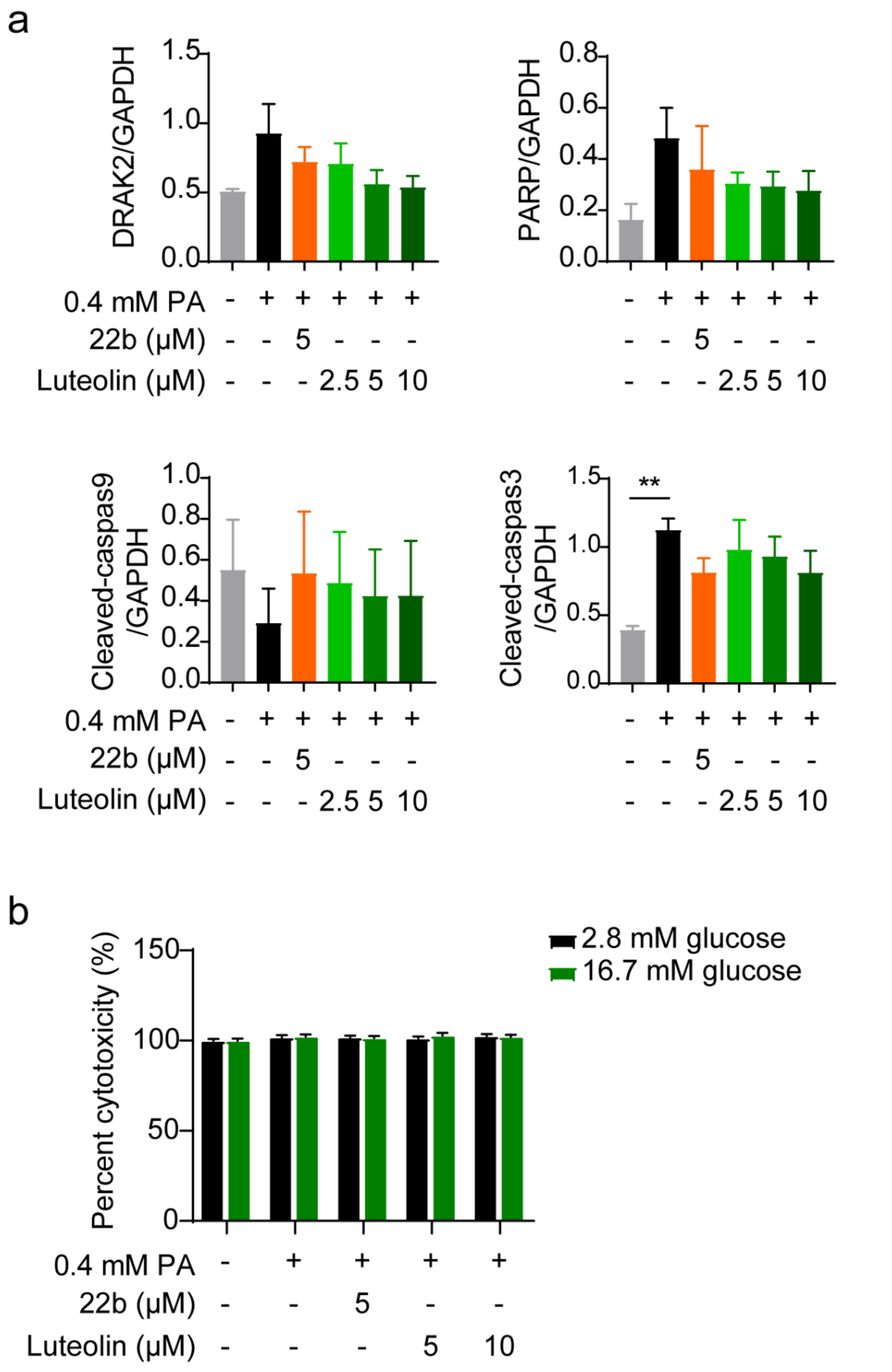


**Figure S1.** ***Related to Figure 2.*** **Contents of LDH in KRBH solution after therapy**

(**a**). Grayscale analysis of Figure 2c, n= 3. (**b**). LDH levels in KRBH solution measured by CytoTox-ONE™ Homogeneous Membrane Integrity Assay, n= 3.

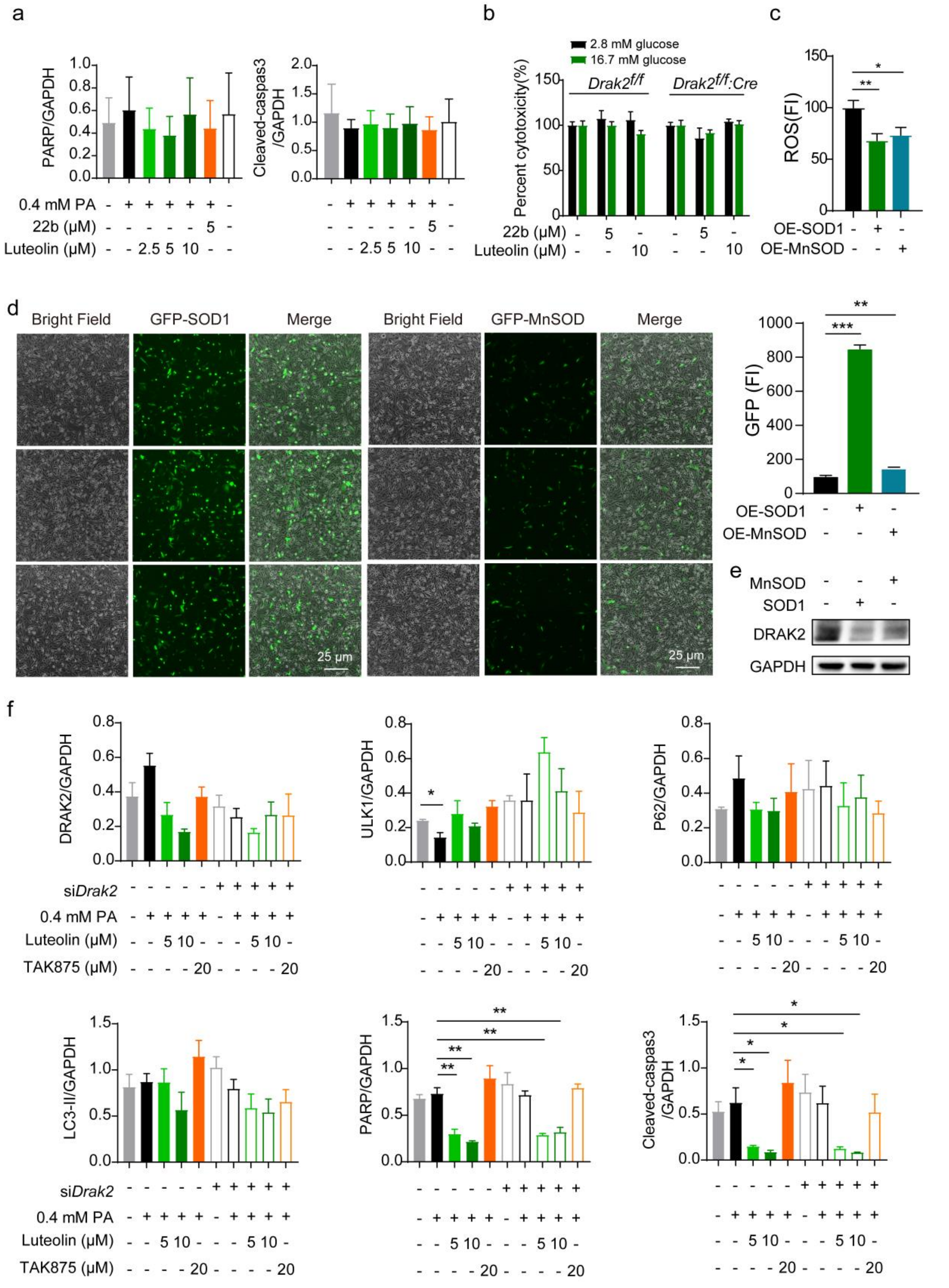


**Figure S2.** ***Related to Figure 3*****. Content of LDH in KRBH solution after therapy and SOD fluorescence intensity in CHO cells**

(**a**). Grayscale analysis of Figure 3a, n= 3. (**b**). LDH levels in KRBH solution measured by CytoTox-ONE™ Homogeneous Membrane Integrity Assay, n= 3. (**c**)-(**d**). Overexpressing SOD1 or MnSOD in CHO cells. (c) The ROS levels in CHO cells, n= 4. (d) Fluorescence intensity and quantification of SOD in CHO cells after overexpressing SOD, n= 6. (**e**). Western-blot analysis of DRAK2 protein levels in response to overexpression of SOD and MnSOD. (**f**). Grayscale analysis of Figure 3d, n= 3. * $P < 0.05$, ** $P < 0.01$, *** $P < 0.001$.

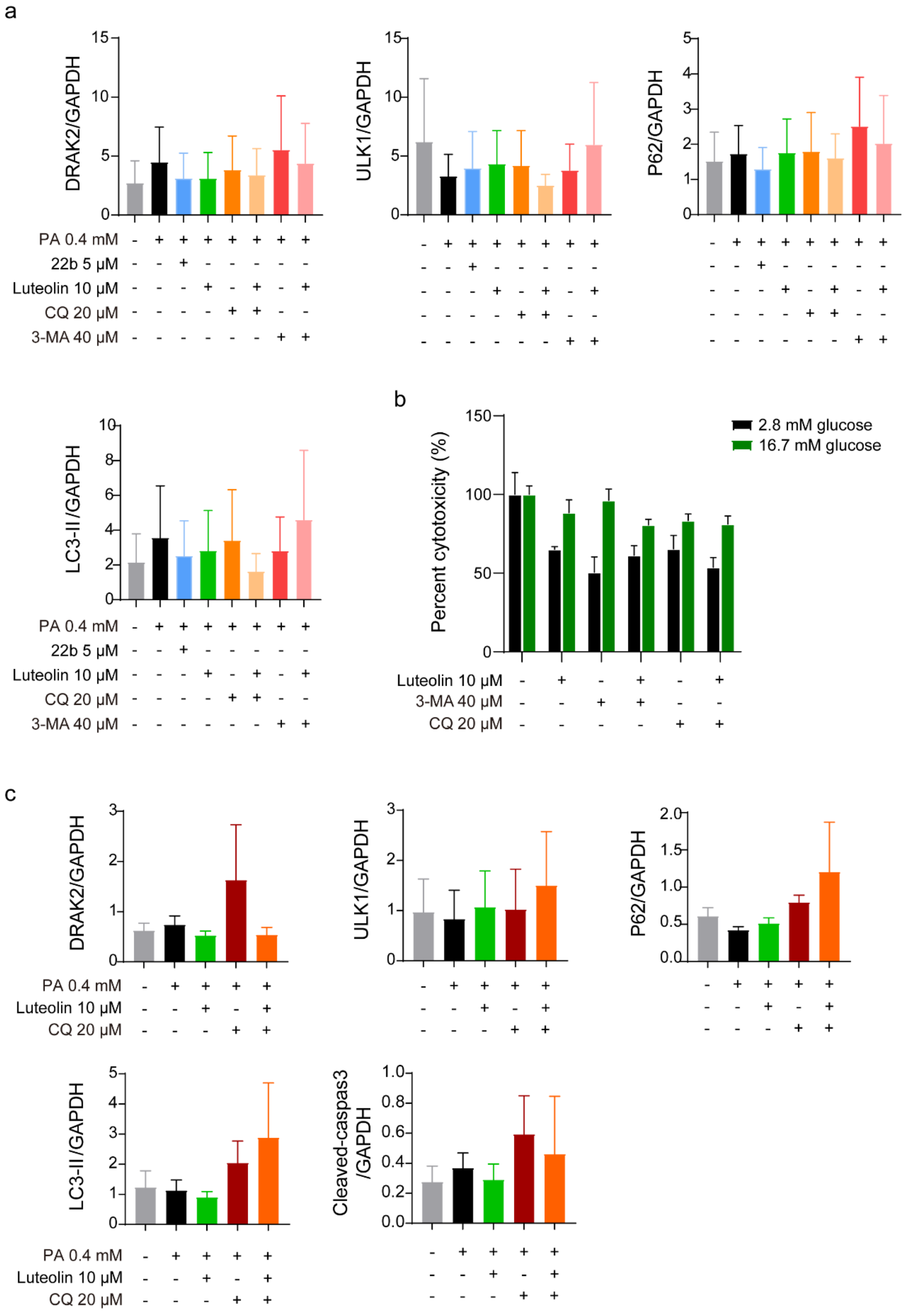


**Figure S3.** ***Related to Figure 4.*** **Content of LDH in KRBH solution after therapy**

(**a**). Grayscale analysis of Figure 4b, n= 3. (**b**). LDH levels in KRBH solution measured by CytoTox-ONE™ Homogeneous Membrane Integrity Assay, n= 3. (**c**). Grayscale analysis of Figure 4d, n= 3.

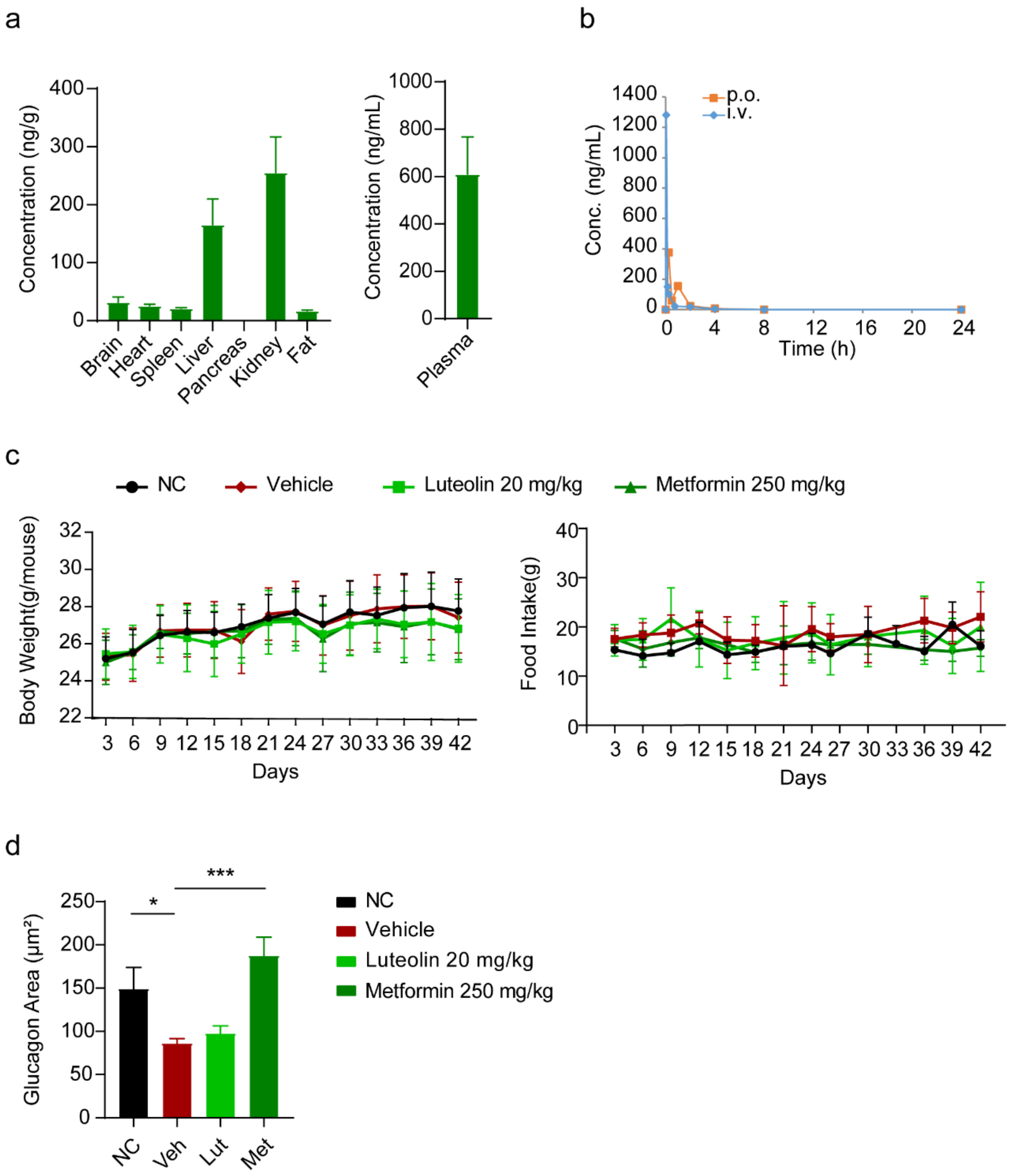


**Figure S4.** ***Related to Figure 5*****. Protective effect of luteolin on mice**

(**a**). Tissue distribution of luteolin by oral on ICR mice, n= 4. (**b**). Drug-time curve of luteolin *in vivo*. (**c**). Body weight on the last day of dosing and the average food intake during the 42 days treatment period, n= 8-13. (**d**). Area of glucagon assessed by immunofluorescence, n= 20.

Table S2. Pharmacokinetics parameters of Luteolin after p.o and i.v. administration in mouse

| Administration | $T_{1/2}$ (h) | $T_{max}$ (h) | $C_{max}$ (ng/mL) | $AUC_{last}$ (h*ng/mL) | $AUC_{INF\_obs}$ (h*ng/mL) | $CL_{\_obs}$ mL/min/kg | $MRT_{INF\_obs}$ (h) | $Vss_{\_obs}$ (mL/kg) | F (%) |
|---|---|---|---|---|---|---|---|---|---|
| p.o. | 0.714 | 0.25 | 376 | 241 | 248 | - | 1.13 | - | 34.4 |
| i.v. | 1.36 | - | - | 175 | 184 | 453 | 0.879 | 23887 | |

Comment: N/A

**Table S2. Related to Figure 5. Concentration-time Data**

The pharmacokinetics parameters of luteolin after p.o. and i.v. administration in ICR mice
